# Supplementary figures and images for: Tiny but complex - interactive 3D visualization of the interstitial acochlidian gastropod Pseudunela cornuta (Challis, 1970)
Source: Front Zool. 2009 Sep 11;6:20. doi: 10.1186/1742-9994-6-20 (PMC2761907; doi:10.1186/1742-9994-6-20)

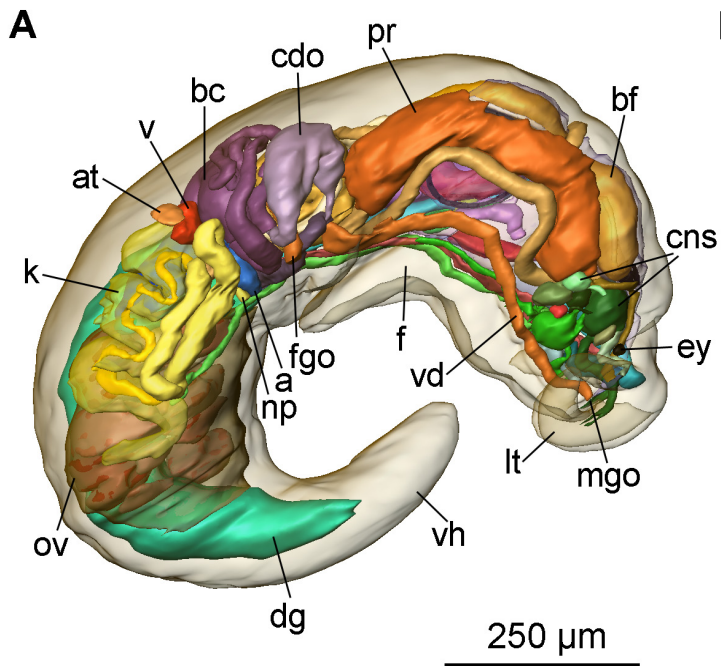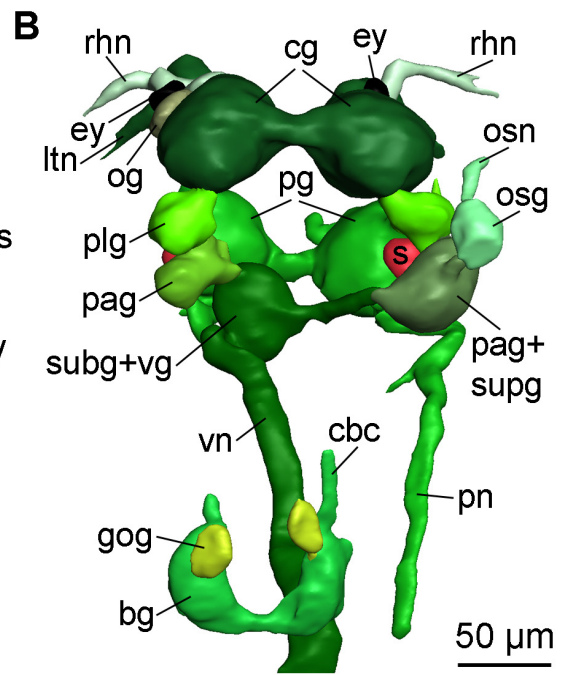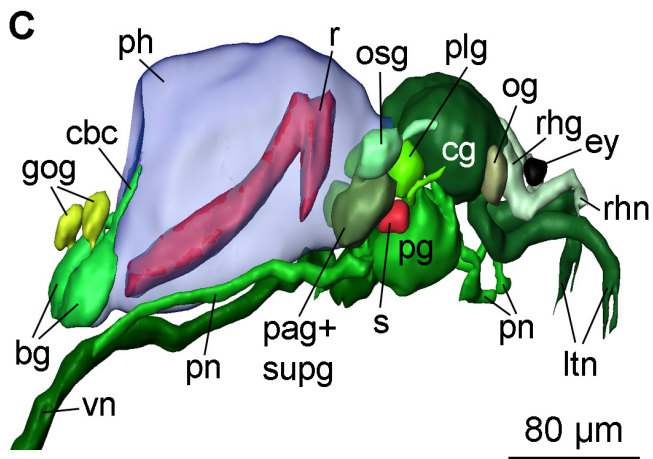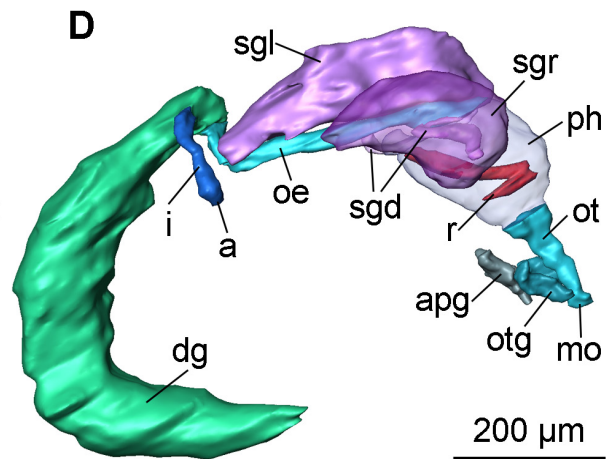

Supplement: Additional file 1 — Interactive 3D-model of Pseudunela cornuta. The file provided includes an interactive 3D-model of the anatomy of Pseudunela cornuta. The interactive 3D-model of P. cornuta can be accessed by clicking into Fig. 1. Rotate model by dragging with left mouse button pressed, shift model: same action + ctrl (or change default action for left mouse button), zoom: use mouse wheel. Select or deselect (or change transparency of) components in the model tree, switch between prefab views or change surface visualization (e.g. lightning, render mode, crop etc.). Interactive manipulation requires Adobe Reader 7 or higher. [file 1742-9994-6-20-S1.pdf]
