# Supplementary material for: Tiny but complex - interactive 3D visualization of the interstitial acochlidian gastropod Pseudunela cornuta (Challis, 1970)
Source: Front Zool. 2009 Sep 11;6:20. doi: 10.1186/1742-9994-6-20 (PMC2761907; doi:10.1186/1742-9994-6-20)
Supplement: Additional file 2 — Pdf file of this article with interactive figure 1 - for details see Additional file 1. [file 1742-9994-6-20-S2.pdf]

Frontiers in Zoology

BioMed Central

Research

Open Access

Tiny but complex - interactive 3D visualization of the interstitial acochlidian gastropod *Pseudunela cornuta* (Challis, 1970)

Timea P Neusser*[1,2], Martin Hess[2] and Michael Schrödl[1,2]

Address: [1]Zoologische Staatssammlung München, Münchhausenstr. 21, 81247 München, Germany and [2]Department Biology I, Ludwig-Maximilians-Universität München, Grosshadernerstr. 2, 82152 Planegg-Martinsried, Germany

Email: Timea P Neusser* - timea-neusser@gmx.de; Martin Hess - hess@bio.lmu.de; Michael Schrödl - Michael.Schroedl@zsm.mwn.de

* Corresponding author

Published: 11 September 2009

Frontiers in Zoology 2009, **6**:20 doi:10.1186/1742-9994-6-20

Received: 12 March 2009

Accepted: 11 September 2009

This article is available from: http://www.frontiersinzoology.com/content/6/1/20

© 2009 Neusser et al; licensee BioMed Central Ltd.

This is an Open Access article distributed under the terms of the Creative Commons Attribution License (http://creativecommons.org/licenses/by/2.0), which permits unrestricted use, distribution, and reproduction in any medium, provided the original work is properly cited.

Abstract

Background: Mesopsammic acochlidians are small, and organ complexity may be strongly reduced (regressive evolution by progenesis), especially in microhedylacean species. The marine interstitial hedylopsacean *Pseudunela cornuta* (Challis, 1970), however, was suggested as having a complex reproductive system resembling that of much larger, limnic and benthic species. The present study aims to reconstruct the detailed anatomy and true complexity of *P. cornuta* from serial, semithin histological sections by using modern computer-based 3D visualization with Amira software, and to explain it in an evolutionary context.

Results: Our results demonstrate considerable discordance with the original species description, which was based solely on paraffin sections. Here, we show that the nervous system of *P. cornuta* has paired rhinophoral, optic and gastro-oesophageal ganglia, three distinct ganglia on the visceral nerve cord, and a putative osphradial ganglion, while anterior accessory ganglia are absent. The presence of an anal genital cloaca is clearly rejected and the anus, nephropore and gonopore open separately to the exterior; the circulatory and excretory systems are well-differentiated, including a two-chambered heart and a complex kidney with a long, looped nephroduct; the special androdiaulic reproductive system shows two allosperm receptacles, three nidamental glands, a cavity with unknown function, as well as highly complex anterior copulatory organs with two separate glandular and impregnatory systems including a penial stylet that measures approximately a third of the whole length of the preserved specimen.

Conclusion: In spite of its small body size, the interstitial hermaphroditic *P. cornuta* shows high complexity regarding all major organ systems; the excretory system is as differentiated as in species of the sister clade, the limnic and much larger Acochlidiidae, and the reproductive system is by far the most elaborated one ever observed in a mesopsammic gastropod, though functionally not yet fully understood. Such organ complexity as shown herein by interactive 3D visualization is not plesiomorphically maintained from a larger, benthic ancestor, but newly evolved within small marine hedylopsacean ancestors of *P. cornuta*. The common picture of general organ regression within mesopsammic acochlidians thus is valid for microhedylacean species only.

Background

The meiofauna of marine sands includes species of nearly all taxa of invertebrates, many of which show regressive characteristics in their anatomy or specialized features in their organ systems [1]. Compared to their supposed basal opisthobranch relatives [2,3], mesopsammic acochlidian sea slugs display many of such reductions, e.g., they have a small and worm-like body, lack a shell, are unpigmented, cephalic tentacles and eyes are reduced in several lineages, many species are aphallic, and in general, the reproductive, excretory and circulatory systems have a very simple organization. Due to such reductions, which are especially pronounced in one subclade, the Microhedylacea, the Acochlidia were hypothesized to have undergone "regressive evolution" [4], as a result of progenesis [5]. However, several recent studies [6,7] show that original, macroscopic or paraffin-based histological descriptions of small acochlidian species could hardly give a reliable picture even of simple organs. In contrast, computer-based 3D-reconstruction of serial semithin histological slices is highly efficient to obtain detailed and reliable knowledge even on tiny and complex structures, such as the considerably differentiated acochlidian central nervous system [8-10].

Species of the second acochlidian subclade, the Hedylopsacea, may show fewer tendencies for reductions; in contrast to the microhedylaceans, the circulatory and excretory systems, and reproductive and copulatory organs may be highly complex and are derived especially in members of the Acochlidiidae s.l., a clade of larger-sized, benthic, limnic members [3]. According to a phylogenetic analysis [3], the genus *Pseudunela* is the sistergroup to such derived acochlidians, despite species of *Pseudunela* being small, marine, interstitial forms. Only two *Pseudunela* species are known, *P. eirene* Wawra, 1988 [11] and *P. cornuta* [12]. The description of *P. eirene* is brief and based on a single specimen with ganglia of the nervous system and stylets of copulatory organs studied on a whole-mount by light microscopy only. No histological sections were made, and the radula was studied light-microscopically after dissolving the soft parts and stylets. Information on other organ systems is absent, and no further specimens are available for study. In contrast, the original description of *P. cornuta*, the type species, is based on paraffin sections, and quite detailed data about the central nervous and the digestive systems is included. However, information about the excretory system is fragmentary and improper, and data about the reproductive system is confusing. Well-preserved specimens of *P. cornuta* were made available for detailed 3D-reconstruction. The present study thus explores the complex anatomy and potential role of a member of the stemgroup of a radiation that accounted for major evolutionary changes, i.e. a habitat switch to freshwater systems and an evolution towards highly complex copulatory systems that culminated in a giant, trap-like "rapto-penis".

Methods

Sampling and specimen preparation

During an expedition to Guadalcanal, Solomon Islands in October 2007, two specimens of *Pseudunela cornuta* were collected at the beach of Komimbo Bay near Tambea Village (09°15.843'S, 159°40.097'E). They were extracted from sand samples (fine sand of the lower intertidal) according to Schrödl [13] and relaxed using 7% $MgCl_2$ solution. Both specimens were preserved in 75% ethanol.

Later in the laboratory, the visceral sac of one specimen was removed for further molecular analysis. The remaining anterior body and the other entire specimen were decalcified with Bouin's solution overnight. For better visibility of the translucent specimens and an appropriate orientation during the embedding procedure, the material was stained with Safranin (0.5% Safranin in 80% ethanol) for a few minutes and rinsed with 80% ethanol. Finally, the two specimens (in one case only anterior part) were dehydrated in a graded series of acetone in distilled water (80, 90 and 100%) and embedded in Spurr's low viscosity resin [14]. Two series of ribboned serial semithin sections of 1.5 μm thickness were prepared using a diamond knife (Histo Jumbo, Diatome, Biel, Switzerland) and contact cement at the lower cutting edge [15], and finally stained with methylene blue-azure II according to Richardson *et al.* [16]. The sections were deposited at the Zoologische Staatssammlung München, Mollusca Section (entire specimen: ZSM N° 20071911 and anterior body: ZSM N° 20071809).

3D reconstruction

Digital photographs of every slice (420 images in total) were taken with a CCD microscope camera (Spot Insight, Diagnostic Instruments, Sterling Heights, USA) mounted on a DMB-RBE microscope (Leica Microsystems, Wetzlar, Germany). The image resolution was reduced to 1120 × 840 pixels (resulting pixel size: 0.8 μm) and images were contrast enhanced, unsharp masked and converted to 8bit greyscale format with standard image editing software. A detailed computer-based 3D-reconstruction of all major organ systems was conducted with the software AMIRA 4.1 and 5.2 (TGS Europe, Mercury Computer Systems, Merignac Cedex, France) following basically the procedure explained by Ruthensteiner [15]. The interactive 3D model for the electronic 3D PDF version were prepared using the 3D tools of Adobe Acrobat Professional Extended 9.0 (Adobe Systems Incorporated) according to Ruthensteiner & Heß [17]. The 3D model (accessible by clicking onto Fig. 1 in the 3D PDF version of this article;

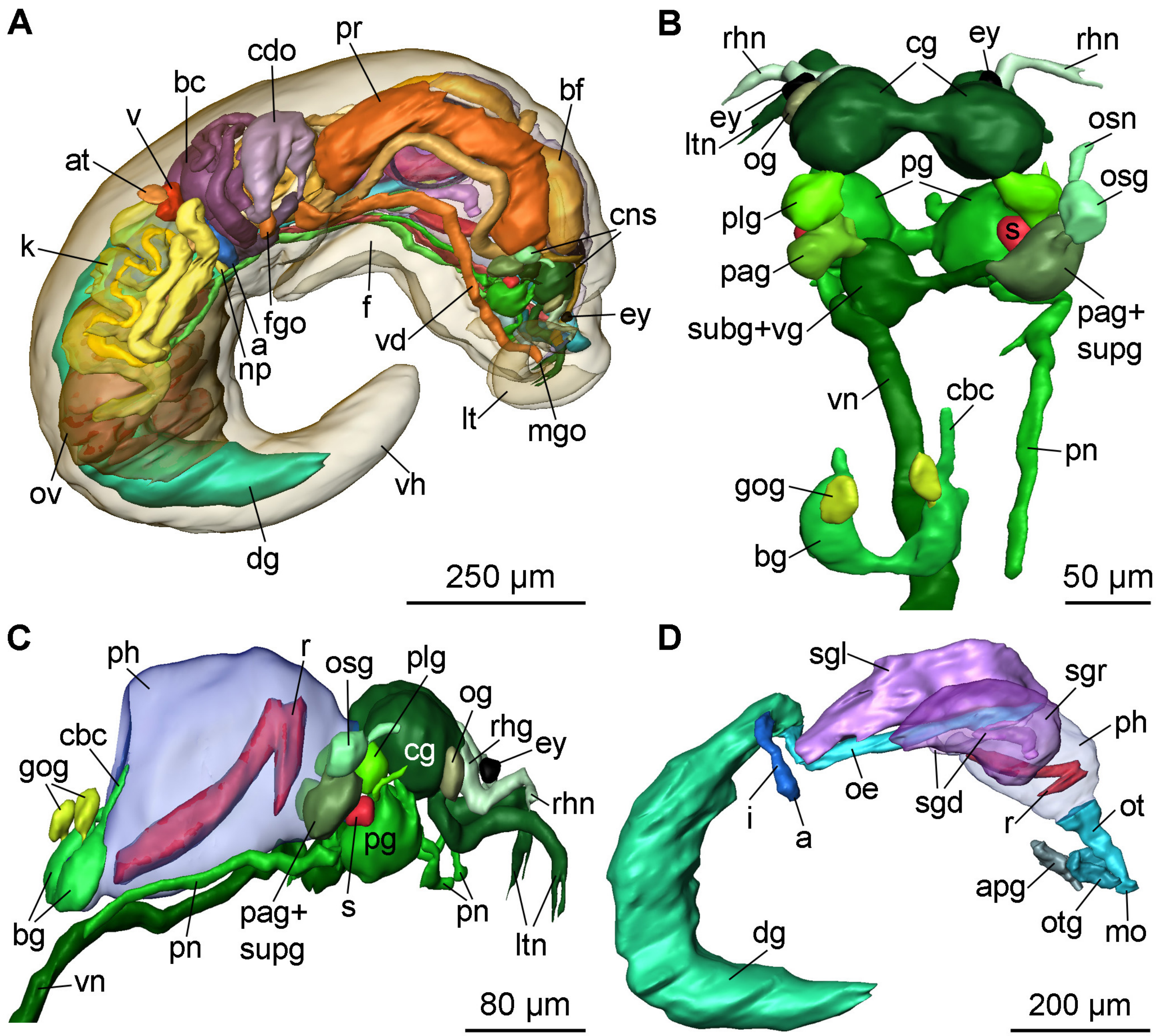

Figure 1
3D reconstruction of the general anatomy, the CNS and the digestive system of *P. cornuta*. A: general anatomy, right view. B: CNS, dorsal view. C: CNS with pharynx, right view. D: digestive system, right view. Abbreviations: **a**, anus; **apg**, anterior pedal gland; **at**, atrium; **bc**, bursa copulatrix; **bf**, basal finger; **bg**, buccal ganglion; **cbc**, cerebro-buccal connective; **cdo**, cavity of distal oviduct; **cg**, cerebral ganglion; **cns**, central nervous system; **dg**, digestive gland; **ey**, eye; **f**, foot; **fgo**, female gonopore; **gog**, gastro-oesophageal ganglion; **i**, intestine; **k**, kidney; **lt**, labial tentacle; **ltn**, labial tentacle nerve; **mgo**, male gonopore; **mo**, mouth opening; **np**, nephropore; **oe**, oesophagus; **og**, optic ganglion; **osg**, osphradial ganglion; **osn**, osphradial nerve; **ot**, oral tube; **otg**, oral tube gland; **ov**, ovotestis; **pag**, parietal ganglion; **pg**, pedal ganglion; **ph**, pharynx; **plg**, pleural ganglion; **pn**, pedal nerve; **pr**, prostate; **r**, radula; **rhg**, rhinophoral ganglion; **rhn**, rhinophoral nerve; **s**, statocyst; **sgd**, salivary gland duct; **sgl**, left salivary gland; **sgr**, right salivary gland; **subg**, subintestinal ganglion; **supg**, supraintestinal ganglion; **v**, ventricle; **vd**, vas deferens; **vg**, visceral ganglion; **vn**, visceral nerve; **vh**, visceral sac. The interactive 3D-model of *P. cornuta* can be accessed by clicking onto Fig. 1 in the 3D PDF version of this article; see also additional file 1 (Adobe Reader Version 7 or higher required). Rotate model by dragging with left mouse button pressed, shift model: same action + ctrl (or change default action for left mouse button), zoom: use mouse wheel. Select or deselect (or change transparency of) components in the model tree, switch between prefab views or change surface visualization (e.g. lightning, render mode, crop etc.).

see also additional file 1) permits standard operations as zoom and rotation, the selection of the reconstructed structures and switching between prefabricated views.

Original material and neotype

According to Challis [12], the holotype of *Pseudunela cornuta*, 20 paratypes and a slide with the radula of a further paratype were deposited in The Natural History Museum, London; furthermore, 10 paratypes and a slide with another radula were deposited in the Museum of New Zealand Te Papa Tongarewa, Wellington; the remaining paratypes and the sectioned material were stored in the private collection. We contacted both museums above mentioned - there is no trace of the material or any evidence that it ever arrived there. Obviously, no type material of *P. cornuta* was ever deposited in any public institution.

We consider our recently collected specimens as the species *Pseudunela cornuta* described by Challis [12] due to 1) the same collecting site as part of the material that was used for the original description, 2) the undoubted placement into the genus *Pseudunela* and 3) the same external morphology as described by Challis [12]. The section series ZSM N° 20071911 is designed herein as neotype due to the apparent non-existence of the original type material, and to avoid taxonomic confusion with congeners and a number of similar but still unnamed species found by the authors and mentioned in the literature [18-20].

Results

The following description is based on the entire specimen, which shows mature reproductive organs.

External morphology

Pseudunela cornuta shows an anterior head-foot complex and a posterior elongated visceral hump (vh) (Figs. 1A; 2) in which the animal can partly retract when disturbed. The paired labial tentacles (lt) (Figs. 1A; 2) are broad at the base, tapering to the end and usually held at 45°-90° to the longitudinal axis of the specimen. The paired rhinophores (rh) (Fig. 2) are tapered and usually point forward like horns in crawling animals. Eyes (ey) are present (Fig. 1A-C), but not visible externally. The densely ciliated foot (f) is as broad as the anterior head-foot complex and extends about one third of the visceral hump in the crawling animal. The free end of the foot is pointed (Fig. 2).

The body size of living specimens is about 3 mm and the body colour is whitish translucent. In the anterior part of the visceral hump the heart bulb (hb) (Fig. 2) is visible externally on the right body side. A few elongate, subepidermal spicules of up to 40 µm in length can be found in the posterior part of the visceral hump.

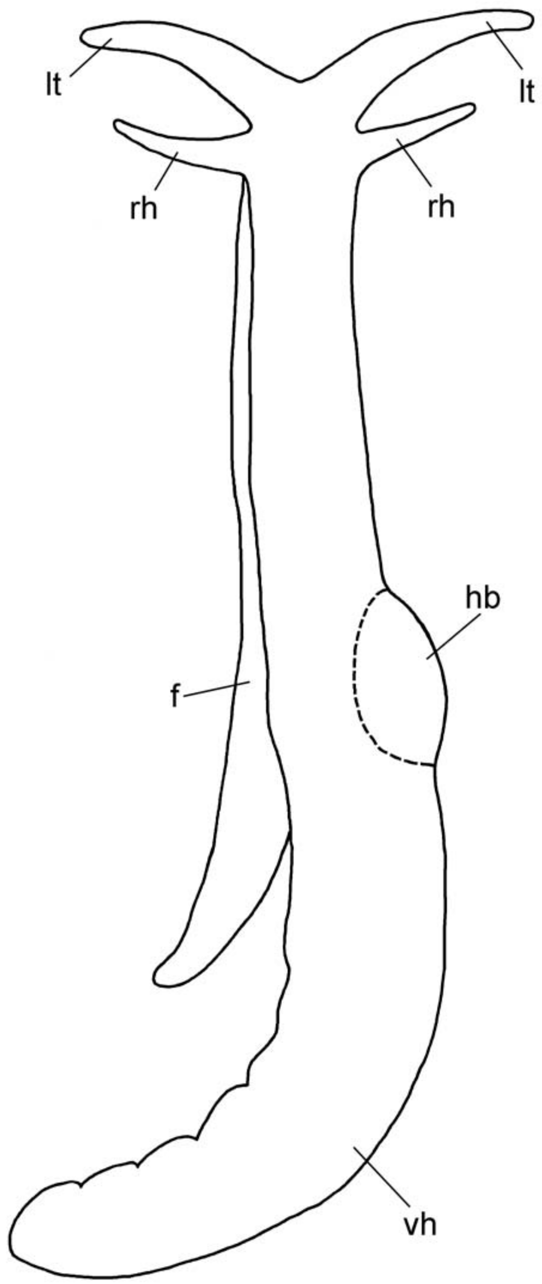

Figure 2
External morphology of *P. cornuta* (schematic drawing, dorsal view). Abbreviations: **f**, foot; **hb**, heart bulb; **lt**, labial tentacle; **rh**, rhinophore; **vh**, visceral hump.

Microanatomy

Central nervous system (CNS)

The CNS of *Pseudunela cornuta* is euthyneurous and composed of the paired cerebral (cg), rhinophoral (rhg), optic (og), pedal (pg), pleural (plg), buccal (bg) and gastro-oesophageal ganglia (gog) as well as three distinct ganglia

on the visceral nerve cord, plus a presumed osphradial ganglion (osg) (Figs. 1B, C; 3). All ganglia excluding the buccal and gastro-oesophageal ganglia are situated pre-pharyngeally (Fig. 1C). The CNS is epiathroid; the pleural ganglion is located closer to the cerebral ganglion than to the pedal one. All ganglia consist of an outer cortex containing the nuclei and an inner medulla (Fig. 4A-C). The large cerebral ganglia are linked by a robust commissure (Figs. 1B; 3) and lie dorsal to the pedal ganglia (Fig. 1C). Anteroventrally, the robust labiotentacular nerve (ltn) (Figs. 1C; 3; 4B) emerges innervating the labial tentacle. A rhinophoral ganglion (Figs. 1C; 3; 4A) is situated antero-dorsally to each cerebral ganglion connected by a short, single cerebro-rhinophoral connective. The rhinophoral nerve (rhn) (Figs. 1B, C; 3) arises from the rhinophoral ganglion extending to the rhinophore. A small, unpigmented eye (Figs. 1A, C; 4A) is connected by the thin optic nerve (on) (Fig. 3) to the rhinophoral nerve, slightly anterior to the rhinophoral ganglion. An optic ganglion (Figs. 1C; 3; 4B) is attached laterally to each cerebral ganglion and connected to the latter by a thin nerve (Fig. 3). The optic ganglion is surrounded by an additional layer of connective tissue shared with the cerebral ganglion. Precerebral anterior accessory ganglia, as described for microhedylacean acochlidians and *Tantulum elegans* Rankin, 1979 [6-8,10], are absent. A Hancock's organ could not be detected.

The paired pedal ganglia (Figs. 1B, C; 3) lie posteroventrally to the cerebral ganglia, and are connected by a commissure which is slightly longer than the cerebral commissure (Figs. 1B; 3). A statocyst (s) with a single otolith (Figs. 1B, C; 3; 4C) is attached dorsally to each pedal ganglion. The static nerve could not be detected. Two pedal nerves (pn) (Figs. 1C; 3) emerge from each pedal ganglion, one in the anterior and another in the posterior part, both innervating the foot. The pleural ganglion is located posterior to the cerebral ganglion (Figs. 1B, C; 3; 4C) and connected to the latter and the pedal ganglion by short connectives forming the pre-pharyngeal nerve ring. The pleural ganglia are connected by very short connectives to the visceral nerve cord, so that the latter is located at the very beginning of the pharynx (Fig. 1C). There are three distinct ganglia on the short visceral nerve cord: the left parietal ganglion (pag) (Figs. 1B; 3; 4C), the fused subintestinal/visceral ganglion (subg+vg) (Figs. 1B; 3; 4C) and the fused right parietal/supraintestinal ganglion (pag+supg) (Figs. 1B, C; 3). While the left pleuro-parietal, the parietal-subintestinal/visceral and the right pleuro-parietal/supraintestinal connectives are very short, the subintestinal/visceral-parietal/supraintestinal connective is long (Fig. 3). An additional presumed osphradial ganglion (Figs. 1B, C; 3) is linked to the fused parietal/supraintestinal ganglion. Anteriorly, a nerve emerges (Figs. 1B; 3; 4C) and innervates the right body wall; no histologically differentiated osphradium could be detected. The buccal ganglia are positioned posterior to the pharynx (Fig. 1C) and are linked to each other by a short buccal commissure ventral to the oesophagus (Fig. 4E). The thin cerebro-buccal connective (Figs. 1B; 3) emerges anteriorly from each buccal ganglion and was not traceable along the entire length. A smaller gastro-oesophageal ganglion (Figs. 1B, C; 3; 4E) lies dorsally to each buccal ganglion and is connected to the latter by a short connective.

Digestive system

The mouth opening (mo) (Fig. 1D) lies ventrally between the labial tentacles. The paired anterior pedal glands (apg) (Figs. 1D; 4A) discharge ventral to the mouth opening to the exterior. The oral tube (ot) (Figs. 1D; 4A, B) is long and not ciliated. Paired oral tube glands (otg) (Figs. 1D; 4A, B) are flanking the oral tube and discharge in its anterior part. The muscular pharynx (ph) (Figs. 1C; 4C, D) is bulbous and narrows to the posterior; it contains the hook-shaped radula (r) (Figs. 1C, D; 4D). The upper ramus is longer than the lower one (Fig. 1C). The radula formula could not be examined. Jaws are absent. The long, ciliated oesophagus (oe) (Figs. 1D; 4E, F) emerges posterodorsally from the pharynx and is flanked by longitudinal muscles. One pair of large salivary glands (sgl, sgr) (Figs. 1D; 4E) discharges into the oesophagus via narrow salivary gland ducts (sgd) (Figs. 1D; 4E) directly behind the pharynx.

The large, sac-like digestive gland (dg) (Fig. 1D) is placed at the left side of the visceral hump flanking the ovotestis (Figs. 1A; 5E) and extends almost up to the end of the visceral hump (Fig. 1A). The intestine (i) is densely ciliated and short (Figs. 1D; 5A, B). The anus (a) (Fig. 1A, D) opens slightly anterior, but separate to the nephropore and ventrolaterally on the right side of the visceral hump.

Excretory and circulatory systems

The excretory and circulatory systems are located at the right side of the body (Fig. 1A) just at the beginning of the visceral hump.

The circulatory system shows a large two-chambered heart consisting of an anterior ventricle (v) (Figs. 1A; 5F; 6; 7A, B) and a smaller, posterior atrium (at) (Figs. 5F; 6; 7A, B). The thin-walled pericardium (pc) (Fig. 6) surrounding the heart could not be detected due to the very compressed tissue. The aorta (ao) (Figs. 5A; 6; 7A, B) arises anteriorly from the ventricle and leads to the head, where the aorta bifurcates (Figs. 4A, B; 6) approximately at the level of the eyes ending in blood sinuses. The renopericardioduct (rpd) (Figs. 6; 7B) is a well-developed and heavily ciliated funnel (Figs. 5B; 6B). The kidney (k) is a sinuously bent sac and extends over almost the half of the visceral hump

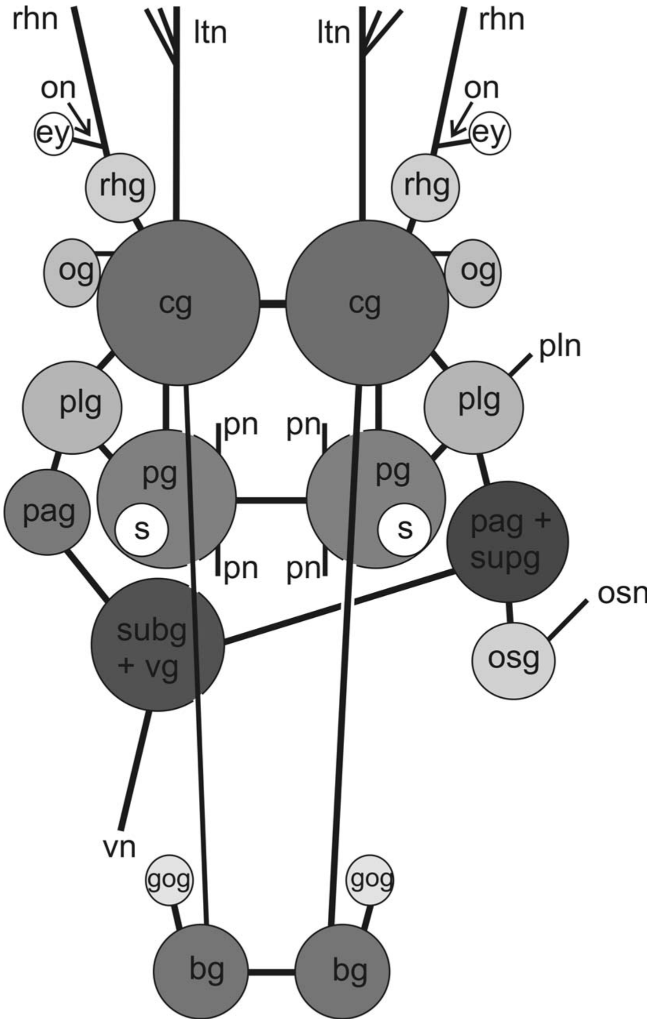

Figure 3
CNS of *P. cornuta* (schematic overview, dorsal view). Abbreviations: **bg**, buccal ganglion; **cg**, cerebral ganglion; **ey**, eye; **gog**, gastro-oesophageal ganglion; **ltn**, labial tentacle nerve; **og**, optic ganglion; **on**, optic nerve; **osg**, osphradial ganglion; **osn**, osphradial nerve; **pag**, parietal ganglion; **pg**, pedal ganglion; **plg**, pleural ganglion; **pln**, pleural nerve; **pn**, pedal nerve; **rhg**, rhinophoral ganglion; **rhn**, rhinophoral nerve; **s**, statocyst; **subg**, subintestinal ganglion; **supg**, supraintestinal ganglion; **vg**, visceral ganglion; **vn**, visceral nerve. Not to scale.

(Fig. 1A). Internally it is divided into a narrow lumen (kn) (Figs. 5D; 6A; 7A, B) bordered by tissue with small vacuoles, and a wide lumen (kw) (Figs. 5C, D; 6; 7A, B) limited by highly vacuolated tissue. Both lumina join in the posterior part of the kidney (Fig. 6). The renopericardial duct is connected to the narrow lumen in the anterior part of the kidney (Figs. 6B; 7B). The connection between the kidney and the nephroduct is narrow and ciliated. The nephroduct is long and looped with a dorsal branch (ndd) extending backward and a ventral branch (ndv) forward (Figs. 6; 7A, B). The ventral branch is looped dorsally in its distal part (Figs. 6; 7A, B). The nephropore (np) (Fig. 1A) opens just posterior, but separate to the anus and ventrolaterally on the right side of the visceral hump.

Reproductive system

Terms used below are based on Ghiselin [21]. The nidamental glands are identified according to Klussmann-Kolb [22] and the anterior male copulatory organs are named following the terminology of Haase & Wawra [23].

The reproductive system of *Pseudunela cornuta* is (simultaneous) hermaphroditic (Fig. 8). The anterior genitalia show a special androdiaulic condition: the vas deferens does not branch off in a proximal position as usual in androdiaulic nudibranch or acteonoidean species [2,24,25], but more distally, i.e. autosperm must pass through the nidamental glands. Nevertheless this reproductive system is not strictly monaulic, because the internal vas deferens (for autosperm) is separated from the distal portion of the oviduct.

The sac-like ovotestis (ov) extends over the half of the right side of the visceral hump (Fig. 1A) and is not separated into follicles; oocytes are located more in the exterior part of the gonad and the spermatocytes are positioned more in the centre. Sperm heads are short (Fig. 5E). Approximately 10 yolky oocytes (oo) were noted in the examined specimen (Figs. 5A, E; 7C). Anterior to the ovotestis there is a small receptaculum seminis (rs) (Figs. 5A, B; 7C; 8) containing sperm cells orientated with their heads to the wall, as well as a sac-like ampulla (am) (Figs. 7C, D; 8) filled with unorientated autosperm (Figs. 4F; 5A). Three nidamental glands can be distinguished: the albumen (alg), membrane (meg) and mucus gland (mug) from proximal to distal, respectively (Figs. 7C, D; 8). The tube-like albumen gland is characterized by cells containing dark blue stained vesicles and long cilia (Fig. 5A-D). The membrane gland is tube-like with long cilia as well. In the proximal part, vesicles are stained purple, in the distal part, lilac (Fig. 5A, D). The mucus gland is sac-like with short cilia. It shows the same histological staining properties as the distal membrane gland (Fig. 5B, D). The distal part of the mucus gland extends to the right side of the body wall where the hermaphroditic duct divides into the vas deferens (vd) and the oviduct (Fig. 8). The oviduct widens to a cavity (cdo) (Figs. 5B-D, F; 7C, D; 8). At the distal end of the cavity a long, narrowly coiled bursa stalk (bs) (Figs. 5B-D, F; 7C, D; 8) branches off leading to the large bursa copulatrix (bc) (Figs. 5D, F; 7C, D; 8). No spermatocytes can be detected inside the bursa, but an indeterminable mucous mass that might contain degenerated sperm. The distal oviduct (do) extends to the female gonopore (fgo) (Figs. 4F; 7C, D; 8) opening ventrolaterally on the right side of the visceral hump to the exterior.

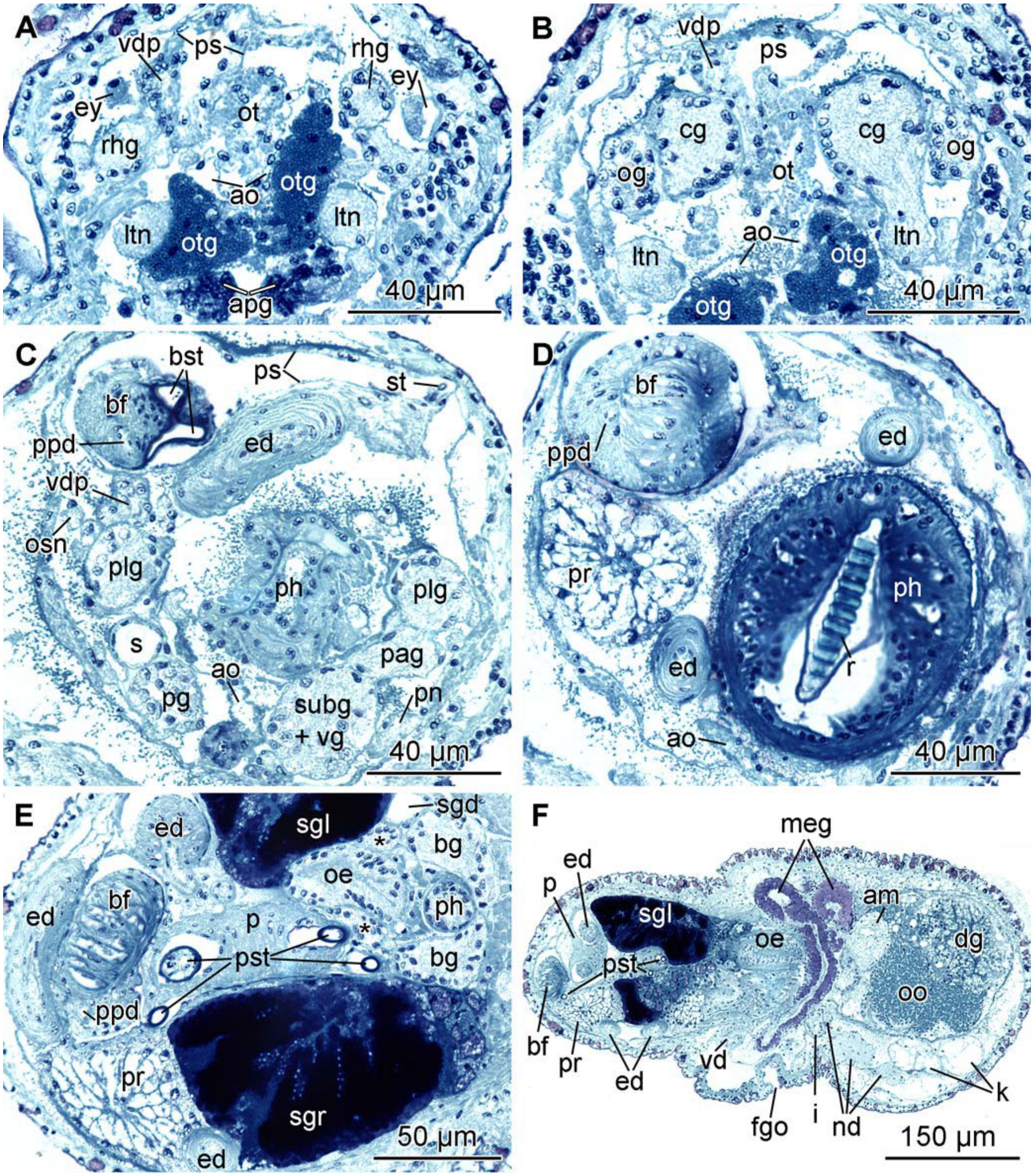

Figure 4

Histological cross-sections of *P. cornuta*. A: eye and rhinophoral ganglion. B: cerebral and optic ganglia. C: pleural, parietal and fused subintestinal/visceral ganglion. D: pharynx and basal finger. E: buccal ganglion and penial stylet. F: female gonopore and membrane gland. Abbreviations: **am**, ampulla; **ao**, aorta; **apg**, anterior pedal gland; **bf**, basal finger; **bg**, buccal ganglion; **bst**, stylet of basal finger (base); **cg**, cerebral ganglion; **dg**, digestive gland; **ed**, ejaculatory duct; **ey**, eye; **fgo**, female gonopore; **i**, intestine; **k**, kidney; **ltn**, labial tentacle nerve; **meg**, membrane gland; **nd**, nephroduct; **oe**, oesophagus; **og**, optic ganglion; **oo**, oocyte; **osn**, osphradial nerve; **ot**, oral tube; **otg**, oral tube gland; **p**, penis; **pag**, parietal ganglion; **pg**, pedal ganglion; **ph**, pharynx; **plg**, pleural ganglion; **pn**, pedal nerve; **ppd**, paraprostatic duct; **pr**, prostate; **ps**, penial sheath; **pst**, penial stylet; **r**, radula; **rhg**, rhinophoral ganglion; **s**, statocyst; **sgd**, salivary gland duct; **sgl**, left salivary gland; **sgr**, right salivary gland; **st**, stylet of basal finger (tip); **subg**, subintestinal ganglion; **vd**, vas deferens; **vdp**, posterior-leading vas deferens; **vg**, visceral ganglion; *****, gastro-oesophageal ganglion.

The female gonopore is situated considerably anterior to the anus and the nephropore (Fig. 1A).

The internal, subepidermal vas deferens extends along the right body side (Figs. 4; 8) to the right rhinophore connecting to the anterior male copulatory organs (Figs. 7E; 8). The short posterior-leading vas deferens (vdp) (Figs. 4B, C; 7C; 8) joins the large, tubular prostate gland (pr) (Figs. 4D, E; 7C, E; 8). Anteriorly, the long and highly coiled, muscular ejaculatory duct (ed) arises from the prostate (Figs. 4C-F; 5A-C; 7C, E; 8). The ejaculatory duct enters the muscular penis (p) (Figs. 4E, F; 7E, F; 8) at its base and discharges at the top of the penis through a long hollow stylet. The penial stylet (pst) is about 600 μm long and corkscrew-like coiled with one and a half spirals (Figs. 4E, F; 7F; 8). This stylet can be partly retracted into the penial muscle (Figs. 4E; 7F) that is able to evert to a certain extent. The blind ending glandular paraprostate (ppr) (Figs. 5A-D; 7E; 8) is longer and thinner than the prostate, and in contrast to the latter, highly coiled. It is connected by the paraprostatic duct (ppd) (Figs. 5A-D; 7E; 8) to the muscular basal finger (bf) (Figs. 4C-F; 7C, E, F; 8), which is united to the penial muscle mass at its base. The paraprostatic duct enters the basal finger approximately in the middle of the muscle (Fig. 7E) and opens terminally via a hollow curved stylet (bst, st) (Figs. 4C; 7E, F; 8) of about 110 μm length. The penis, the basal finger and parts of the ejaculatory and paraprostatic ducts are surrounded by a thin-walled penial sheath (ps) (Figs. 4C; 7E, F; 8). The latter, together with the copulatory organs, probably can be protruded through the male gonopore (mgo) (Fig. 1A) just at the base of the right rhinophore during the sperm transfer. However, sperm transfer has never been observed in living specimens.

Discussion

External morphology

The body of *Pseudunela cornuta* is divided into an anterior head-foot complex and the elongated visceral hump, as characteristic for Acochlidia [3]. The digitiform shape and the position of the cephalic tentacles identify this species as belonging to the genus *Pseudunela*, according to Salvini-Plawen [26], Rankin [27] and Wawra [28]. Our results of the external morphology match with the original description of Challis [12], except for the presence of subepidermal spicules in living specimens. Most probably Challis overlooked the sparsely arranged spicules in the visceral hump of *P. cornuta* or they were already dissolved in preserved specimens.

Microanatomy

Central nervous system

Challis' original description of the CNS in *Pseudunela cornuta* contains some substantial details [12]. In the present study we supplement and correct the original data, and, in addition, homologize and name ganglia according to standard works [29]. The ganglia on the visceral nerve cord were interpreted according to the pentaganglionate hypothesis proposed by Haszprunar and recent studies on other acochlidians [6,30,31].

The CNS of *P. cornuta* follows the usual arrangement of ganglia in other hedylopsacean acochlidian species such as *Hedylopsis ballantinei* Sommerfeldt & Schrödl, 2005 and *Tantulum elegans* [6,31]. In contrast to *T. elegans*, precerebral ganglia are lacking in *P. cornuta*. Challis [12] described precerebral anterior accessory ganglia for *P. cornuta* as "anterior nerves in the form of two chains of ganglia". According to the drawing in Challis [12], the highly undulated and curled nerves might have been misinterpreted as anterior accessory ganglia. Anterior accessory ganglia are absent in a recently discovered congener from Vanuatu [32], but have been reported for *P. eirene* by Wawra [11] and, thus, should be re-examined carefully in this species.

Although Challis [12] described some very tiny nerves, such as the static nerve and the cerebro-buccal connectives, he overlooked or misinterpreted quite larger structures, such as the paired rhinophoral, optic and gastro-oesophageal ganglia. Our results show the eye is innervated by the optic nerve which emerges from the rhinophoral nerve; this condition is very unusual for opisthobranch species and, to our knowledge, only known for the closely related acochlidians *Hedylopsis ballantinei* and *H. spiculifera* (Kowalevsky, 1901) [31,33]. In contrast, the eye in the more basal *Tantulum elegans* is innervated by the optic nerve arising from the optic ganglion; additionally, the optic nerve is connected to the Hancock's nerve [6]. Challis [12] described only two ganglia on the visceral nerve cord, namely the sub- and the supraintestinal ganglia, which are identified in the present work as the fused subintestinal/visceral and the fused right parietal/supraintestinal ganglion, respectively. The small left parietal ganglion has been overlooked, probably due to its very close position to the pleural ganglion. The additional ganglion attached to the fused parietal/supraintestinal ganglion, which has been described originally as visceral ganglion [12], is interpreted herein as the osphradial ganglion, according to Huber [29].

Digestive system

The digestive system of *Pseudunela cornuta* was well-described by Challis [12] and conforms to the general ground-pattern of the digestive system in acochlidian species. The stomach reported in the original description, however, could not be detected in the present study. While a stomach fused with the anterior cavity of the

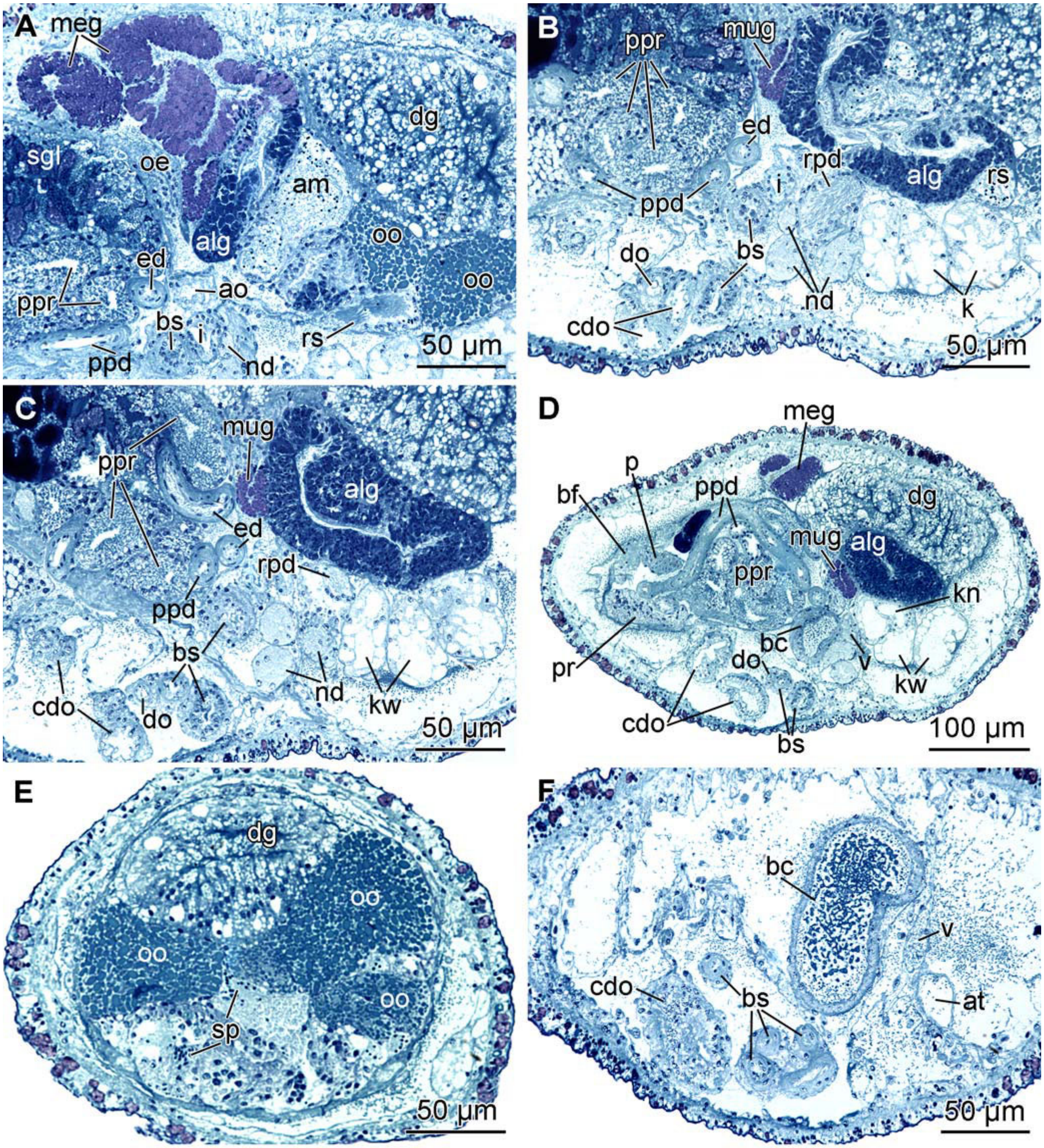

Figure 5

Histological cross-sections of *P. cornuta*. A: ampulla and receptaculum seminis. B: renopericardioduct. C: albumen gland. D: paraprostate. E: ovotestis with oocytes and spermatocytes. F: bursa copulatrix and atrium. Abbreviations: **alg**, albumen gland; **am**, ampulla; **ao**, aorta; **at**, atrium; **bc**, bursa copulatrix; **bf**, basal finger; **bs**, bursa stalk; **cdo**, cavity of distal oviduct; **dg**, digestive gland; **do**, distal oviduct; **ed**, ejaculatory duct; **i**, intestine; **k**, kidney; **kn**, narrow lumen of kidney; **kw**, wide lumen of kidney; **meg**, membrane gland; **mug**, mucus gland; **nd**, nephroduct; **oe**, oesophagus; **oo**, oocytes; **p**, penis; **ppd**, paraprostatic duct; **ppr**, paraprostate; **pr**, prostate; **rpd**, renopericardioduct; **rs**, receptaculum seminis; **sgl**, left salivary gland; **sp**, spermatocytes; **v**, ventricle.

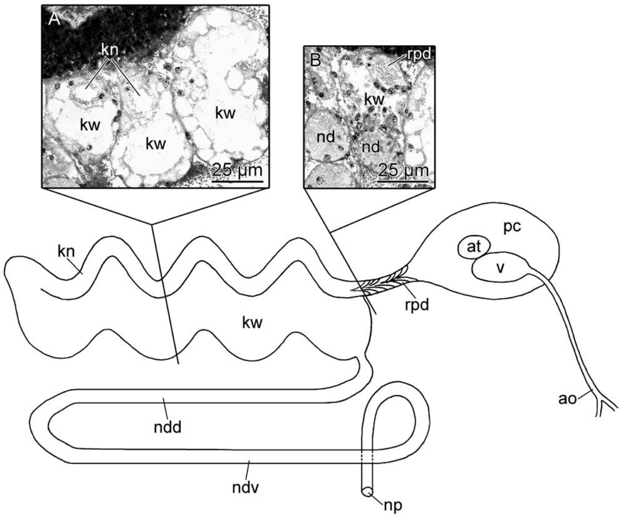

Figure 6
Circulatory and excretory systems of *P. cornuta* (schematic drawing, right view and histological cross-sections). A: narrow and wide lumen of kidney. B: transition of renopericardioduct and kidney. Abbreviations: **ao**, aorta; **at**, atrium; **kn**, narrow lumen of kidney; **kw**, wide lumen of kidney; **ndd**, dorsal branch of nephroduct; **ndv**, ventral branch of nephroduct; **np**, nephropore; **pc**, pericardium; **rpd**, renopericardioduct; **v**, ventricle. Not to scale.

digestive gland is present in some acochlidian species, such as *T. elegans* and *Asperspina murmanica* (Kudinskaya & Minichev, 1978) [6,7], a histologically and anatomically distinct organ is absent in all Acochlidia studied in detail.

Acochlidians generally have reduced or lost the mantle cavity. While in *Hedylopsis ballantinei* a small remainder could be detected by histological and ultrastructural investigations [34], a well-developed "mantle-cavity" originally described from *A. murmanica* was shown to be completely absent [7,35]; the genital system, intestine and nephroduct open separately at the right lateral body surface [7]. The presence of common exit ducts, such as cloacae, could indicate that there are remnants of mantle cavities in some acochlidians. Challis described an anal-genital cloaca into which the intestine is discharging from *P. cornuta*; however, this assumption is clearly rejected by our results. In *P. cornuta* the genital opening, anus and nephropore open separately to the exterior (from anterior to posterior, respectively). Additionally, the anus is associated with the nephropore; the female gonopore opens more anteriorly. The same arrangement of the orifices of the body can be found in *T. elegans* [6], whereas the nephropore is situated anterior to the anus in the microhedylacean *Microhedyle remanei* (Marcus, 1953), *A. murmanica* and *Pontohedyle milaschewitchii* (Kowalevsky, 1901) [7,8,10]. Another acochlidian species, *Asperspina rhopalotecta* (Salvini-Plawen, 1973), which was reported to show a true cloaca [28], should be re-examined carefully.

Excretory and circulatory systems
The excretory and circulatory systems of *P. cornuta* were rudimentarily described by Challis who identified a pericardium, a heart without evident division into ventricle and atrium, and a short aorta "discharging almost immediately into the haemocoele" [12]. In contrast, our results show a two-chambered heart and an aorta extending up to the head. Well-developed two-chambered hearts have been reported for *Hedylopsis ballantinei*, *Microhedyle remanei* and *Tantulum elegans* [6,8,34]. In contrast, only a one-chambered heart could be detected recently in *Asperspina murmanica* and *Pontohedyle milaschewitchii* in spite of detailed re-examinations [7,10]. Jörger *et al.* [10] suggest a thorough examination by TEM for all acochlidian species reported with a one-chambered heart or described as being even heart-less, such as *Ganitus evelinae* Marcus, 1953 and *Parhedyle tyrtowii* (Kowalevsky, 1901) [36,37].

The kidney of *P. cornuta* has been depicted as a "large unfolded sac" [12] without any internal and histological data given. Surprisingly, our present data reveal that the kidney is a large, complex organ showing histologically distinguishable sections with supposedly different, but yet unknown function. In contrast, all marine acochlidian species studied in detail (*M. remanei*, *P. milaschewitchii* and *A. murmanica*) have a small, simple, sac-like kidney [7,8,10]. The marine *Hedylopsis ballantinei* was reported to show a long, sac-like kidney extending almost over the entire visceral sac [31,34]; however, our re-examination revealed a complex kidney with a narrow duct extending posteriorly and a wide one leading anteriorly (own unpubl. data), just as in *P. cornuta*. The kidney of *P. cornuta* also resembles those described for limnic hedylopsaceans such as *T. elegans* [6]. The original description of *P. cornuta* does not provide any information about the length and the shape of the nephroduct, nor the position of the nephropore. Whereas marine acochlidian species usually have a short, straight nephroduct (such as *M. remanei*, *P. milaschewitchii*, *A. murmanica*), the present study reveals *P. cornuta* to have a long, looped nephroduct as present in limnic Acochlidiidae (own unpubl. data) [38].

Unfortunately, Wawra [11] did not mention any excretory or circulatory features in the description of *Pseudunela eirene*, thus no comparison to other *Pseudunela* species can be drawn.

Reproductive system
The original description of the genital organs [12] shows major discrepancies relative to our results. Besides revising

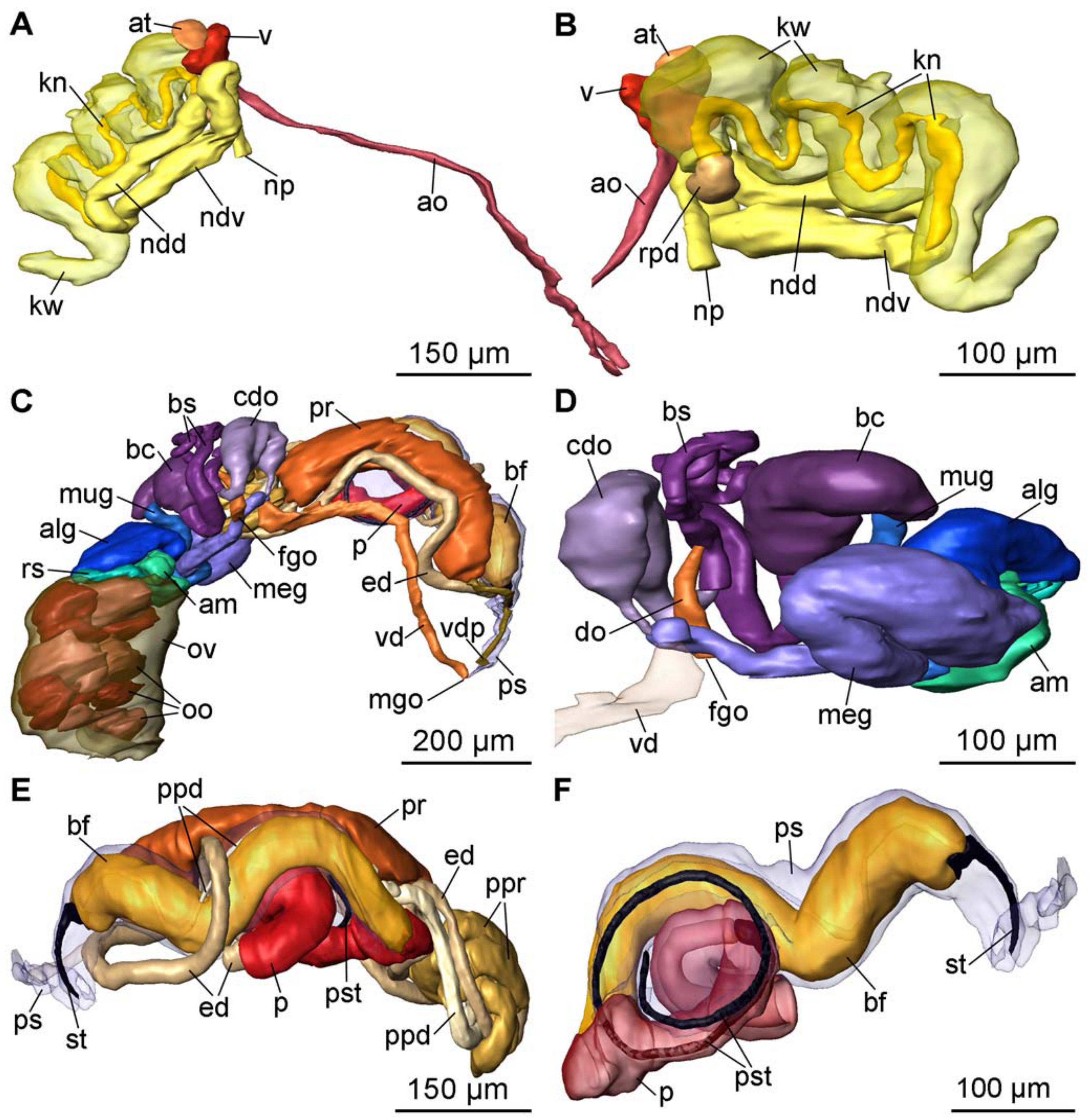

Figure 7

3D reconstruction of the excretory and circulatory systems and the reproductive system of *P. cornuta*. A: circulatory and excretory systems, right view. B: circulatory and excretory systems, left view. C: complete reproductive system, right view. D: nidamental glands and sperm storing receptacles, left view. E: anterior male copulatory organs, left view. F: penis and basal finger, anterolaterally right view. Abbreviations: **alg**, albumen gland; **am**, ampulla; **ao**, aorta; **at**, atrium; **bc**, bursa copulatrix; **bf**, basal finger; **bs**, bursa stalk; **cdo**, cavity of distal oviduct; **do**, distal oviduct; **ed**, ejaculatory duct; **fgo**, female gonopore; **kn**, narrow lumen of kidney; **kw**, wide lumen of kidney; **meg**, membrane gland; **mgo**, male gonopore; **mug**, mucus gland; **ndd**, dorsal branch of nephroduct; **ndv**, ventral branch of nephroduct; **np**, nephropore; **oo**, oocyte; **ov**, ovotestis; **p**, penis; **ppd**, paraprostatic duct; **ppr**, paraprostate; **pr**, prostate; **ps**, penial sheath; **pst**, penial stylet; **rpd**, renopericardioduct; **rs**, receptaculum seminis; **st**, stylet of basal finger; **v**, ventricle; **vd**, vas deferens; **vdp**, posterior-leading vas deferens.

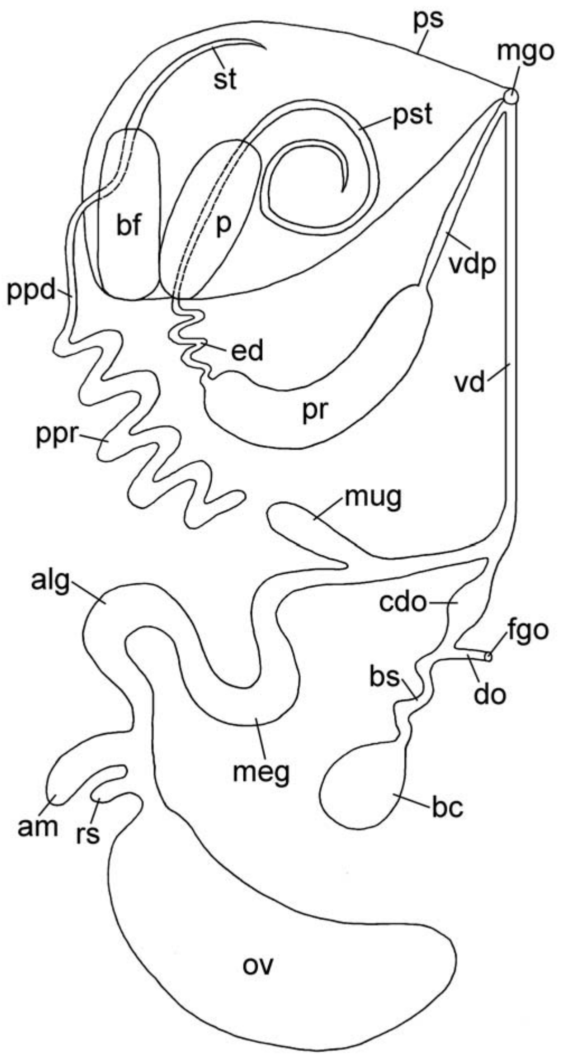

Figure 8
Reproductive system of *P. cornuta* (schematic drawing, dorsal view). Abbreviations: **alg**, albumen gland; **am**, ampulla; **bc**, bursa copulatrix; **bf**, basal finger; **bs**, bursa stalk; **cdo**, cavity of distal oviduct; **do**, distal oviduct; **ed**, ejaculatory duct; **fgo**, female gonopore; **meg**, membrane gland; **mgo**, male gonopore; **mug**, mucus gland; **ov**, ovotestis; **p**, penis; **ppd**, paraprostatic duct; **ppr**, paraprostate; **pr**, prostate; **ps**, penial sheath; **pst**, penial stylet; **rs**, receptaculum seminis; **st**, stylet of basal finger; **vd**, vas deferens; **vdp**, posterior-leading vas deferens. Not to scale.

the differences, we add new data and name structures according to Haase & Wawra [23].

The reproductive system of the opisthobranch common ancestor likely was monaulic and the pallial gonoduct undivided [21]. Most acochlidian species may have a monaulic reproductive system as well (or are gonochoristic). In contrast, a special type of an androdiaulic reproductive system with the distal portion of the female gonoduct separated from the vas deferens exists in *Pseudunela cornuta* and *Tantulum elegans* [6]. Challis [12] noticed the presence of a distal bursa copulatrix as a short blind sac emerging from the "cloaca", but, in contrast to our observations, there is no report of a proximally situated receptaculum seminis. In the past, only the limnic acochlidian *Strubellia paradoxa* (Strubell, 1892) from Solomon Islands was known to possess both allosperm receptacles [39]. While in the original description no ampulla was described, we could find a well-developed, sac-like ampulla in *P. cornuta*. A sac-like ampulla is reported from *Asperspina murmanica* and *Tantulum elegans* [6,7], whereas the ampulla is a tubular swelling of the gonoduct in *Microhedyle remanei* and *Pontohedyle milaschewitchii* [8,10]. Opisthobranch eggs are surrounded by three layers of nutritive and protective materials that are secreted by three different glands [21]. Challis described two nidamental glands, the proximal albumen and the distal mucous gland, but gave no data about their shapes or histological appearances. Following Klussmann-Kolb [22], the nidamental glands in this study were interpreted based on their position in the reproductive system. These are the albumen, membrane and mucus gland, from proximal to distal, respectively. The albumen and membrane glands are tubular in all acochlidian species studied in detail. The mucus gland shows more structural variety and may be tubular as in *A. murmanica* and *P. milaschewitchii* [7,10], but is a blind sac in *P. cornuta* and *M. remanei* [8]. The cavity of the distal oviduct in *P. cornuta* that is situated near to the female gonopore was not described by Challis [12] and has never been observed in any other acochlidian species up to now. The function of this structure is yet unknown. A function as fertilization chamber is not likely due to its very distal position in the reproductive system. However, a role during sperm transfer is imaginable (see below).

The posterior part of the reproductive system is connected to the anterior male reproductive system by the completely internal vas deferens. According to Ghiselin [21] the latter is a mechanism to hasten the transfer of sperm and, therefore, is an improvement compared with the external sperm groove of the hypothetic ancestor of the opisthobranchs.

The original description of the complex, anterior copulatory organs includes a drawing by Challis [12]; unfortunately, the interpretation of the different ducts, glands and stylets remains confusing. Wawra [11] interpreted the penial spine in Challis' drawing as the penial stylet. In contrast, we consider herein the penial spine of 100 μm in fact being the stylet of the basal finger (which measures approx. 110 μm in our specimen), so that the following

conclusions can be drawn: 1) the stylet-bearing muscle at the base of the penis in Challis' drawing is the basal finger; 2) the penial gland was misinterpreted and is in fact the paraprostate; 3) the duct connecting Challis' penial gland with the penial spine is considered as the paraprostatic duct; 4) the prostate gland is the prostate; 5) the spermatic duct running from the rhinophore to the prostate gland is the cephalic, posterior-leading vas deferens; 6) the efferent male duct probably is the penial sheath through which the anterior male copulatory organs can be protruded. Furthermore, the ejaculatory duct connecting the prostate to the penis was overlooked, as well as the large hollow stylet that we found at the tip of the penis. May be the stylet was totally retracted into the penial muscle in the specimen examined by Challis, or perhaps it was broken away during the last sperm transfer. Wawra [40] suggested this possibility for *Hedylopsis spiculifera*, as he found a detached stylet in the visceral sac of one specimen. The extremely complex copulatory system found in *P. cornuta* is similar to that of species of the much larger, limnic Acochlidiidae, and particularly the genus *Strubellia* (own unpubl. data).

Reproductive functions

While the generally marine microhedylacean species are aphallic, the basal, limnic hedylopsacean, *Tantulum elegans*, possesses a muscular copulatory organ [6]. Similar, but more complex anterior copulatory organs can be found in the marine hedylopsaceans *Hedylopsis spiculifera*, *Pseudunela cornuta* and *P. eirene* [11,40], as well as in other, limnic hedylopsacean species. The hollow penial stylet of all these latter species indicates that sperm transfer occurs by injection [3,41]. Hypodermal injection in the sequential hermaphrodite *H. spiculifera*, which lacks any allosperm receptacles, may be an imprecise one, as indicated by the finding of lost penial stylets in the body cavity [40]. In *P. cornuta*, we found an extremely long, tubular penial stylet and two allosperm storing receptacles. Due to the presence of the latter, we suggest a more precise sperm injection in *P. cornuta* into the genital system of the mate. In the present species, the cavity of the distal oviduct may serve as the site of sperm injection, or any other place within the genital system. Injected sperm then would move to the receptaculum seminis for long term storage and/or to the bursa copulatrix for short term storage and digestion. Passing through the nidamental glands without being trapped is obviously possible, presumably during periods without active glandular secretion. Challis proposed either the bursa stalk or the cloaca as region of fertilization in *P. cornuta*. This is unlikely due to the absence of the cloaca and the position of the bursa stalk distal to the nidamental glands. Fertilization of oocytes certainly occurs proximally, close to the receptaculum seminis, where allosperm is stored and nourished as indicated by the heads that are embedded into the organ walls.

Peculiar and noteworthy is the very long and curled, hollow penial stylet in *P. cornuta*. While other *Pseudunela* species have a penial stylet not exceeding 200 μm, the penial stylet of *P. cornuta* is approx. 600 μm long, which represents nearly one third of the body length in the fixed specimen. The functionality of such a curled stylet, however, is not understood. The curl may be a fixation artefact or more likely, due to the immense length of the stylet and the little space available in the head, the curled position signifies a "space saving storage". During sperm transfer the stylet may be uncoiled due to the pressure of emergent fluids and be operative for "long distance" hypodermal impregnation; in this case, the specimen can inject autosperm without approximating too closely the mate and thus, without the risk of being "hit" by the mate. Since the stylet in its extended condition measures over 2 times the complete body width of a potential mate, we cannot imagine of any basic functional needs for developing such an organ, such as injection of sperm into a certain organ or body region of the mate. Instead, we may be observing the product of an evolutionary race of arms within *P. cornuta*. Similarly obscure is the exact function of an additional, paraprostatic impregnatory system that was described from *Acochlidium fijiense* Haynes & Kenchington, 1991 [23]. Schrödl & Neusser [3] discussed a probable role in the production of anaesthetics as known in cephalaspidean species with complex penial structures [42] or of fluids stimulating sperm transfer, as known from the sacoglossan *Elysia timida* (Risso, 1818) [43]. In *P. cornuta*, however, the penial stylet is extremely long, and it is difficult to imagine how the much shorter stylet of the basal finger may hit and affect the mate.

Regression or innovation? Evolution of acochlidian organ systems

Based on our recent results on acochlidian phylogeny [3], the evolution of organs and whole organ systems can be reconstructed at least for the major clades. In contrast to earlier generalizations [4,5], the various lineages show different trends; an overview of reductions and increasing complexity of the organ systems in Acochlidia is given in Fig. 9. The topology of the phylogenetic tree (parsimony analysis for all nominal 27 acochlidian species and 11 outgroup taxa based on 107 morphological characters) is simplified according to Schrödl & Neusser [3].

The external morphology with the anterior head-foot complex retractile into an elongated visceral hump is similar in all acochlidian species and certainly ancestral. Only in the microhedylacean species is there a tendency towards reduction of the cephalic tentacles, the foot length and the foot width (Fig. 9), whereas *P. cornuta*

shows, together with all other hedylopsacean species, well-developed tentacles and foot. The digestive system of *P. cornuta* is quite simple and conforms to the usual ground-pattern in acochlidian species. The CNS is plesiomorphically complex and the arrangement of ganglia is more or less similar in all acochlidian species. Differences concern precerebral accessory ganglia which, after splitting off *Tantulum*, were lost in the hedylopsacean lineage (Fig. 9), still by marine ancestors. In contrast, aggregations of accessory ganglia are present in microhedylacean species. The acochlidian excretory system varies considerably between marine and limnic species. All microhedylacean species known in detail show a small, simple and sac-like kidney and a short nephroduct [7,8,10]. While members of *Hedylopsis* were reported to have a simple, but long kidney [31,34,44], our re-examination of *Hedylopsis ballantinei* showed this species having a complex, bent kidney, as well (own unpubl. data). Since this special type of kidney seems present in all Hedylopsacea (Fig. 9), but neither in microhedylacean acochlidians nor in potential outgroup taxa, we propose that it has evolved in the mesopsammic ancestor of hedylopsaceans. This organ thus is of marine origin, still occurs in marine species and is equally structured in limnic species such as the basal, small Caribbean *Tantulum elegans* and members of the more derived, large Acochlidiidae that inhabit rivers of tropical Pacific islands. The hedylopsacean kidney thus is assumed to be a preadaptation and key feature to both, independent invasions of a limnic habitat known from opisthobranchs. The evolution of excretory systems and the invasion of freshwater systems in acochlidians clearly merit further study.

The most variable organ system within the Acochlidia is the reproductive system. Lacking any sperm storage or copulatory organs, the latter is considerably reduced from a usual basal opisthobranch condition in all microhedylacean species [3,45]. In contrast, the special androdiaulic genital system of *P. cornuta* with highly elaborated cephalic copulatory organs is clearly more complex than that assumed for the basal opisthobranch acochlidian ancestors. In fact, the hedylopsacean topology as revealed by Schrödl & Neusser [3] points towards the successively increasing complexity of the copulatory system of hypodermal injectors in the hedylopsacean stem line. This is confirmed herein (Fig. 9). The basal *T. elegans* lacks any stylet on the penial muscle and sperm transfer occurs probably by copulation [6]. *Hedylopsis spiculifera* shows a single penial stylet for sperm transfer [40]. While *H. ballantinei* was described to potentially being aphallic [31], we could detect two copulatory stylets or thorns in this species (own unpubl. data); details must be explored in a future study. In contrast, *P. cornuta* has an additional paraprostatic glandular system connected to another stylet (Fig. 9). This is similar to the condition in *Strubellia* (own unpubl. data), the most basal known member of Acochlidiidae. Schrödl & Neusser [3] assume that the function of this accessory impregnation system might be the production of special fluids to enforce unilateral insemination or stimulate sperm transfer. Thus, it might be to the best advantage for each individual being the first in injecting its own sperm and other fluids. Finally, the evolution of complex copulatory organs peaks in the so-called giant "rapto-penis" [3] of *Acochlidium* and *Palliohedyle* (Fig. 9). A schematic overview of the different penial structures is given in Schrödl & Neusser [3].

An increasing complexity of excretory and reproductive organs that evolved in the hedylopsacean stemline already in the mesopsammon (Fig. 9) clearly contradicts Swedmark's [4] hypothesis of a general evolutionary regression in marine mesopsammic acochlidians.

But what are the reasons for the remarkable reduction of the reproductive system in microhedylacean species on the one hand and an otherwise increasing complexity in hedylopsacean species on the other hand? Recently, Jörger *et al.* [45] pointed out that the spatially limited interstitial environment may favour unidirectional sperm transfer while quickly passing by. In basally still hermaphroditic microhedylaceans this occurs by means of spermatophores, dermal insemination (spermatophores are placed somewhere on the body surface) and dermal fertilization (allosperm penetrate the body wall and migrate to the gonad for fertilization). Unidirectional sperm transfer, together with the reduction of the copulatory system might have been prerequisites for the evolution of gonochorism in the ancestor of Microhedylidae s.l., and they all may have been key features for the successful radiation of microhedylacean species [3]. Both the hypothetical acochlidian ancestor and the most basal known hedylopsacean offshoot, *Tantulum elegans*, still use copulation for sperm transfer. Since the latter species is a sequential hermaphrodite, sperm transfer is unilateral; this is, thus, the ancestral condition for acochlidians (Fig. 9). According to our data, unidirectional hypodermal impregnation within the Acochlidia was established in the still mesopsammic hedylopsacean lineage (Fig. 9); first in its most simple form as expressed by *Hedylopsis spiculifera*. Comparisons with other, non-mesopsammic opisthobranchs (e.g. Sacoglossa, Nudibranchia) using hypodermal impregnation [43], will show whether or not an already unilateral mode of sperm transfer may be a precondition for evolving hypodermal impregnation systems. Once established, this more or less quick and violent mode of sperm transfer grants for a selective advantage for injectors. Consequently, along the hedylopsacean stem lineage, more and more sophisticated sperm and auxiliary injection systems,

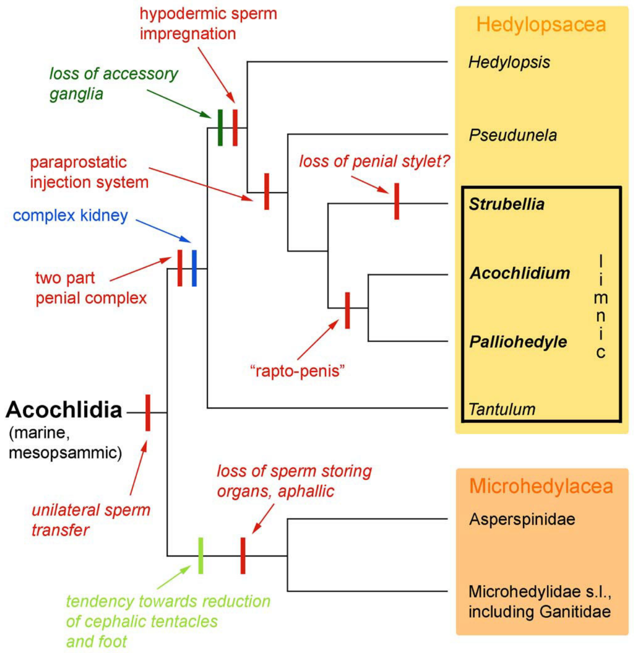

Figure 9
Evolution of organ complexity in acochlidian lineages. A selection of major organ reductions or innovations of several systems is mapped on a phylogenetic tree (strict consensus tree from Schrödl & Neusser [3], simplified. The parsimony analysis was based on 107 morphological characters with all 27 valid acochlidian species and 11 outgroup taxa included). Within the basally marine mesopsammic Hedylopsacea, the reproductive and excretory systems evolved towards higher complexity. With current state of knowledge the special hedylopsacean kidney appears ancestral and can be interpreted as a preadaptation and key feature to successful invasions of freshwater habitats. In contrast, the microhedylacean lineage shows regressive tendencies, especially with regard to external and reproductive features. Light green: external morphology. Dark green: central nervous system. Blue: excretory system. Red: reproductive system. Features in *italic* are reductions/losses, taxa in **bold** refer to large, benthic members of the Acochlidiidae according to Schrödl & Neusser [3].

such as very long penial and accessory paraprostatic stylets in *P. cornuta*, have evolved already in marine mesopsammic environments (Fig. 9). These are similarly retained by the benthic limnic *Strubellia*, but were elaborated into the even more complex and potentially harmful copulatory systems with a giant, armed "rapto-penis" [3] in the ancestor of an array of large-sized benthic, limnic *Acochlidium* and *Palliohedyle* species (Fig. 9), which are no more such spatially limited in their habitat.

Conclusion

Although miniaturization and reductions of organs are characteristic for many interstitial acochlidian species [4], *P. cornuta* shows a complex and complete set of organ systems in spite of the small body size. Remarkable is the high complexity of reproductive organs that resembles that of species of the much larger, limnic Acochlidiidae, and especially the genus *Strubellia*. Unexpectedly, the elaborated excretory system of the marine *P. cornuta* also resembles that of limnic hedylopsacean acochlidians, such as *Tantulum* and Acochlidiidae; the looped kidney and nephroduct are interpreted as evolutionary preadaptations that contributed to successful invasions of limnic systems within the otherwise generally marine Opisthobranchia. Structurally, *Pseudunela cornuta* thus links basal marine with basal and derived limnic clades, reflecting its recently proposed position on the acochlidian tree [3]. Importantly, organ complexity as seen in *P. cornuta* (regarding excretory and reproductive features, at least) is not plesiomorphically retained from a larger, benthic ancestor, but represents innovations that evolved in small, mesopsammic marine acochlidians. Earlier general statements on regressive, progenetic evolution in acochlidians may be relevant for explaining the origin of Acochlidia or that of microhedylacean lineages; *P. cornuta*, however, definitely is an example for evolution of a wealth of sophisticated structures within hedylopsaceans, the exact function of some of which, such as the extremely long spiral penial stylet, still cannot be explained.

Challis' achievement of a quite detailed description has to be acknowledged, since it was almost impossible to describe the complexity of the reproductive system of *P. cornuta* in detail without modern methods. This study once again shows that semithin-histology combined with computer-based 3D reconstruction is highly recommendable for studying the anatomy of micromolluscs, especially for obtaining reliable results that can be used for phylogenetic analyses. An interactive way of publishing 3D models even more impressively demonstrates the complexity of organs in tiny specimens - in the accurate dimensions, positions and relations.

Competing interests

The authors declare that they have no competing interests.

Authors' contributions

TPN carried out the morphological analyses and drafted a manuscript version that was discussed and improved jointly. MH and TPN prepared the interactive 3D model. MS planned and supervised the study. All authors read and approved the final manuscript.

Additional material

Additional file 1

***Interactive 3D-model of* Pseudunela cornuta**. *The file provided includes an interactive 3D-model of the anatomy of* Pseudunela cornuta. *The interactive 3D-model of* P. cornuta *can be accessed by clicking into Fig. 1. Rotate model by dragging with left mouse button pressed, shift model: same action + ctrl (or change default action for left mouse button), zoom: use mouse wheel. Select or deselect (or change transparency of) components in the model tree, switch between prefab views or change surface visualization (e.g. lightning, render mode, crop etc.). Interactive manipulation requires Adobe Reader 7 or higher.*
Click here for file
[http://www.biomedcentral.com/content/supplementary/1742-9994-6-20-S1.pdf]

Acknowledgements

The Natural History Museum, London and the Museum of New Zealand Te Papa Tongarewa, Wellington, provided valuable information about the original material. Katharina Jörger (ZSM) is kindly thanked for collecting the specimens. 3D reconstruction was supported by the GeoBioCenter/LMU München. This study was financed by a grant of the German Research Foundation (DFG SCHR 667/4 to MS). We express thanks to Rick Hochberg (Univ. of Massachussets, USA) and two anonymous reviewers for valuable comments on the manuscript.

References

1. Swedmark B: **The biology of interstitial Mollusca.** *Symp zool Soc Lond* 1968, **22:**135-149.
2. Vonnemann V, Schrödl M, Klussmann-Kolb A, Wägele H: **Reconstruction of the phylogeny of the Opisthobranchia (Mollusca: Gastropoda) by means of 18S and 28S rRNA gene sequences.** *J Moll Stud* 2005, **71:**113-125.
3. Schrödl M, Neusser TP: **Towards a phylogeny and evolution of Acochlidia (Mollusca: Gastropoda: Opisthobranchia).** *Zool J Linn Soc* in press.
4. Swedmark B: **A review of Gastropoda, Brachiopoda, and Echinodermata in marine meiobenthos.** In *Proceedings of the first international conference on Meiofauna Volume 76*. Hulings NC: Smithson Contr Zool; 1971:41-45.
5. Westheide W: **Progenesis as a principle in meiofauna evolution.** *J Nat Hist* 1987, **21:**843-854.
6. Neusser TP, Schrödl M: ***Tantulum elegans* reloaded: a computer-based 3D-visualization of the anatomy of a Caribbean freshwater acochlidian gastropod.** *Invertebr Biol* 2007, **126:**18-39.
7. Neusser TP, Martynov AV, Schrödl M: **Heartless and primitive? 3D reconstruction of the polar acochlidian gastropod *Asperspina murmanica*.** *Acta Zool* 2009, **90:**228-245.
8. Neusser TP, Haszprunar G, Heß M, Schrödl M: **Computerbased 3-dimensional reconstruction of the anatomy of *Microhedyle remanei* (Marcus, 1953), an interstitial acochlidian gastropod from Bermuda.** *J Morphol* 2006, **267:**231-247.
9. Jörger KM, Neusser TP, Schrödl M: **Re-description of a female *Pontohedyle brasilensis* (Rankin, 1979), a junior synonym of the Mediterranean *P. milaschewitchii* (Kowalevsky, 1901) (Acochlidia, Gastropoda).** *Bonn zool Beitr* 2007, **55:**283-290.
10. Jörger KM, Neusser TP, Haszprunar G, Schrödl M: **Undersized and underestimated: 3D-visualization of the Mediterranean interstitial acochlidian gastropod *Pontohedyle milaschewitchii* (Kowalevsky, 1901).** *Org Divers Evol* 2008, **8:**194-214.
11. Wawra E: **Sand-Opisthobranchia aus dem Golf von Bengalen.** *Ann Nathist Mus Wien* 1988, **90:**427-431.
12. Challis DA: ***Hedylopsis cornuta* and *Microhedyle verrucosa*, two new Acochlidiacea (Mollusca: Opisthobranchia) from the Solomon Islands Protectorate.** *Trans R Soc NZ, Biol Sci* 1970, **12:**29-40.
13. Schrödl M: **Techniques for collecting interstitial opisthobranchs.** 2006 [http://www.seaslugforum.net/factsheet.cfm?base=inteextr]. Sea Slug Forum. Australian Museum, Sydney
14. Spurr AR: **A low-viscosity epoxy resin embedding medium for electron microscopy.** *J Ultrastructural Research* 1969, **26:**31-43.
15. Ruthensteiner B: **Soft Part 3D visualization by serial sectioning and computer reconstruction.** *Zoosymposia* 2008, **1:**63-100.
16. Richardson KC, Jarett L, Finke EH: **Embedding in epoxy resins for ultrathin sectioning in electron microscopy.** *Stain Technology* 1960, **35:**313-323.
17. Ruthensteiner B, Heß M: **Embedding 3D models of biological specimens in pdf publications.** *Microsc Research and Technique* 2008, **71:**778-786.
18. Morse P: **Distribution and ecological adaptations of interstitial molluscs on Fiji.** *Amer Malacol Bull* 1987, **5:**281-286.
19. Hughes H: **Sand-dwelling opisthobranchs from Hong Kong.** *J Moll Stud* 1991, **57:**425-431.
20. Hamatani I: **Subclass Opisthobranchia - order Acochlidiacea.** In *Marine Mollusks in Japan* Edited by: Okutani T. Tokyo, Tokai University Press; 2000:760-761.
21. Ghiselin MT: **Reproductive function and the phylogeny of opisthobranch gastropods.** *Malacologia* 1966, **3:**327-378.
22. Klussmann-Kolb A: **Comparative investigation of the genital systems in the Opisthobranchia (Mollusca, Gastropoda) with special emphasis on the nidamental glandular system.** *Zoomorphology* 2001, **120:**215-235.
23. Haase M, Wawra E: **The genital system of *Acochlidium fijiense* (Opisthobranchia: Acochlidioidea) and its inferred function.** *Malacologia* 1996, **38:**143-151.
24. Schmekel L, Portmann A: *Opisthobranchia des Mittelmeeres. Nudibranchia und Saccoglossa* Berlin: Springer-Verlag; 1982.
25. Wägele H, Willan RC: **Phylogeny of the Nudibranchia.** *Zool* 2000, **130:**83-181.
26. Salvini-Plawen L: **Zur Kenntnis der Philinoglossacea und der Acochlidiacea mit Platyhedylidae fam. nov. (Gastropoda, Cephalaspidea).** *Z zool Syst Evolutionsforsch* 1973, **11:**110-133.
27. Rankin JJ: **A freshwater shell-less Mollusc from the Carribean: structure, biotics, and contribution to a new understanding of the Acochlidioidea.** *Life Sci Contrib R Ontario Mus* 1979, **116:**1-123.
28. Wawra E: **Zur Anatomie einiger Acochlidia (Gastropoda, Opisthobranchia) mit einer vorläufigen Revision des Systems und einem Anhang über Platyhedylidae (Opisthobranchia, Ascoglossa).** In *PhD thesis* University Vienna, Dissertation Nr. 17335; 1987.
29. Huber : **On the cerebral nervous system of marine Heterobranchia (Gastropoda).** *J Molluscan Stud* 1993, **59:**381-420.
30. Haszprunar G: **The Heterobranchia - a new concept of the phylogeny of the higher Gastropoda.** *Z f zool Systematik u Evolutionsforschung* 1985, **23:**15-37.
31. Sommerfeldt N, Schrödl M: **Microanatomy of *Hedylopsis ballantinei*, a new interstitial acochlidian gastropod from the Red Sea, and its significance for phylogeny.** *J Moll Stud* 2005, **71:**153-165.
32. Neusser TP, Schrödl M: **Between Vanuatu tides: 3D anatomical reconstruction of a new brackish water acochlidian gastropod from Espiritu Santo.** *Zoosystema* 2009 in press.
33. Neusser TP, Jörger KM, Schrödl M: **Exploring cerebral features in Acochlidia (Gastropoda: Opisthobranchia).** *Bonn zool Beitr* 2007, **55:**301-310.
34. Fahrner A, Haszprunar G: **Microanatomy, ultrastructure, and systematic significance of the excretory system and mantle cavity of an acochlidian gastropod (Opiasthobranchia).** *J Moll Stud* 2002, **68:**87-94.
35. Kudinskaya EV, Minichev YS: **Psammological essays. I. The organization and systematic position of the mollusc *Hedylopsis murmanica* n. sp. (Opisthobranchia, Acochlidiida).** *Trudy Petergofskogo Biologicheskogo Instituta* 1978, **26:**69-86.
36. Kowalevsky A: **Les Hedylidés, étude anatomique.** *Zap Imp Akad Nauk* 1901, **12:**1-32.
37. Marcus E: **Three brazilian sand-opistobranchia.** *Bol Fac Filos, Ci Letr Univ Sao Paulo Zool* 1953, **164:**165-203.
38. Küthe P: **Organisation und systematische Stellung des *Acochlidium paradoxum* STRUBELL.** *Zool Jb Syst* 1935, **66:**513-540.

39. Wawra E: ***Strubellia paradoxa* (Strubell 1892) (Gastropoda: Opisthobranchia) von den Solomon-Inseln.** *Zool Anz* 1988, **220:**163-172.
40. Wawra E: **Zur Kenntnis der interstitiellen Opisthobranchier-art *Hedylopsis spiculifera* (Kowalevsky) (Mollusca, Gastropoda).** *Zool Scr* 1989, **18:**397-403.
41. Wawra E: **Sperm transfer in Acochlidiacea.** *Abstracts of the 11th International Malacological Congress, Siena, University of Siena* 1992:103.
42. Anthes N, Michiels NK: **Precopulatory stabbing, hypodermic injections and unilateral copulations in a hermaphroditic sea slug.** *Biol Lett* 2007, **3:**121-124.
43. Schmitt V, Anthes N, Michiels NK: **Mating behaviour in the sea slug *Elysia timida* (Opisthobranchia, Sacoglossa): hypodermic injection, sperm transfer and balanced reciprocity.** *Front Zool* 2007, **4:**17.
44. Odhner N: ***Hedylopsis suecica* n. sp. und die Nacktschnecken-gruppe Acochlidiacea (Hedylacea).** *Zool Anz (Jena)* 1937, **120:**51-64.
45. Jörger KM, Heß M, Neusser TP, Schrödl M: **Sex in the beach: spermatophores, dermal insemination and 3D sperm ultrastructure of the aphallic mesopsammic *Pontohedyle milaschewitchii* (Acochlidia, Opisthobranchia, Gastropoda).** *Mar Biol* 2009, **156:**1159-1170.

Publish with **BioMed Central** and every scientist can read your work free of charge

"BioMed Central will be the most significant development for disseminating the results of biomedical research in our lifetime."

Sir Paul Nurse, Cancer Research UK

Your research papers will be:

- available free of charge to the entire biomedical community
- peer reviewed and published immediately upon acceptance
- cited in PubMed and archived on PubMed Central
- yours — you keep the copyright

Submit your manuscript here:
http://www.biomedcentral.com/info/publishing_adv.asp

BioMedcentral
